# Medical Imaging Data Calls for a Thoughtful and Collaborative Approach to Data Governance

**DOI:** 10.1371/journal.pdig.0001046

**Published:** 2025-10-28

**Authors:** Aline Lutz de Araujo, Jie Wu, Hugh Harvey, Matthew P. Lungren, Mackenzie Graham, Tim Leiner, Martin J. Willemink

**Affiliations:** 1 Segmed, Inc, Palo Alto, California, United States of America; 2 Hardian Health, Haywards Heath, United Kingdom; 3 Microsoft, Redmond, Washington, United States of America; 4 Department of Radiology, Stanford University School of Medicine, Stanford, California, United States of America; 5 Ethox Centre University of Oxford, Oxford, United Kingdom; 6 Department of Radiology, Mayo Clinic, Rochester, Minnesota, United States of America; CHOP: The Children's Hospital of Philadelphia, UNITED STATES OF AMERICA

## Abstract

The availability of medical imaging data is indispensable for medical advancements such as the development of new diagnostic tools, improved surgical navigation systems, and profiling for personalized medicine through imaging biomarkers. A central challenge in data governance is balancing the need to protect patient privacy with the necessity of promoting scientific innovation. Restrictive data governance policies could limit access to the large, high-quality datasets needed for such advancements. Conversely, lenient policies could compromise patient trust and lead to potential misuse of sensitive information. We call for a deliberate and well-considered approach to data governance, highlighting important factors that patients and healthcare organizations should consider when making imaging data governance decisions around data sharing.

Medical imaging data has unique properties and brings their own challenges to data governance. Data governance in healthcare refers to processes and mechanisms designed to ensure the responsible and secure management of health data throughout its lifecycle [1]. This encompasses the collection, storage, sharing, and use of data within healthcare organizations and beyond, including its secondary use in research and public health initiatives. The main goals of data governance are to protect patient privacy, maintain data integrity, establish compliance with regulations, and promote the ethical use of health data to enable care delivery and advance medical research. The unique challenges associated with imaging data arise from its storage in Picture Archiving and Communication Systems (PACS) rather than Electronic Health Records (EHR). This distinction requires specialized technical capabilities for extracting data from PACS, handling the large file sizes typical of medical images, and applying appropriate de-identification processes to ensure privacy. Beyond these technical considerations, imaging data governance faces additional complexity due to the richness of its associated metadata, which can include acquisition parameters, device identifiers, and embedded patient demographics. Such metadata, while essential for clinical interpretation and research reproducibility, can inadvertently contain or reveal identifiable information. Imaging workflows also involve a broad range of stakeholders—from technologists capturing the images to radiologists producing annotations, referring physicians integrating findings into patient care, and IT teams managing storage and access—each with governance responsibilities and dependencies. Moreover, elements such as radiologist annotations may contain free-text clinical impressions, while device-specific artifacts can indirectly disclose acquisition sites or equipment models, both of which require careful governance oversight. In this opinion piece, we aim to discuss the challenges of data governance related to medical imaging and offer our perspective on how to balance data privacy and security with the need to enable innovation through the secondary use of imaging data.

The primary use of health data is to deliver care to the person from whom the data was collected. Medical images such as radiology and nuclear medicine exams have their primary use directly tied to patient diagnosis, treatment, and monitoring, thus providing immediate clinical value in various clinical scenarios. Once medical imaging data has served its primary purpose, it typically remains stored in PACS and can be used for secondary purposes. Effective data governance must allow leveraging data to improve people’s health by supporting research and healthcare innovations, all while maintaining trust and protecting the rights of individuals. For this to be truly effective, governance frameworks must also recognize the long-term potential of the data.

Imaging data may hold retrospective value over years, enabling future research into disease progression or response to treatment. Conventional governance frameworks, typically focused on short-term data use, may not account for the long-term stewardship and accessibility needed for imaging data. Particularly in the field of artificial intelligence, training and validation of radiological computer-aided detection (CADe) systems predominantly rely on retrospective datasets. Studies supporting pre-market regulatory clearance also use retrospective data, with proven acceptance by regulatory agencies [2]. Examples of medical advancements enabled by imaging datasets include AI software for stroke triage, which reduces radiologist turnaround time and enables earlier intervention, and a fracture detection tool that improves the sensitivity of identifying bone fractures on X-rays [3, 4].

The increasing value of health data for secondary use has sparked debate around data ownership. In traditional contexts, ownership implies a clear delineation of rights and responsibilities; however, in the case of health data, defining ownership is far more complex. Various stakeholders contribute to data generation at different stages — patients, clinicians, allied healthcare professionals, healthcare institutions, and medical device and technology providers — all of whom may have claims to ownership. Framing data sharing solely within the concept of individual ownership is insufficient to address the complexities of modern healthcare. Although patients may wish to exert control over their data, true individual control is impractical and risks placing an undue burden on individuals to protect their data from misuse. Furthermore, the broader public health benefits derived from large-scale datasets may not be realized if individuals retain restrictive control over their data. In light of these challenges, a robust and collaborative governance framework is necessary to balance individual rights with the collective benefits of data sharing.

Robust data governance mechanisms are essential for enabling the use of imaging data for secondary research while protecting patient privacy. Current governance frameworks for imaging data sharing are based on key principles common to other health data types: de-identification of patient data, secure data storage, and opt-in or opt-out protocols that allow patients to control their health data. The availability of opt-in or opt-out choices varies depending on local laws and health provider standards. In contrast, de-identification has become a standard procedure before data sharing, though it presents unique challenges for imaging data. De-identification processes remove several identifiers and usually replace them with unique codes, so that the data can be re-identified or tracked back to the source institutions if necessary. As a result, de-identification considerably reduces, but does not eliminate, the possibility of re-identification. In the United States, the Health Insurance Portability and Accountability Act (HIPAA) defines which pieces of information are potential patient identifiers, also referred to as Protected Health Information (PHI) [5]. Internationally, similar protections exist under frameworks such as the European Union’s General Data Protection Regulation (GDPR), and Canada’s Personal Information Protection and Electronic Documents Act (PIPEDA). While all three frameworks aim to safeguard personal data, GDPR applies to a broader range of personal information and emphasizes explicit consent and the right to erasure (“right to be forgotten”), whereas HIPAA is specific to health information handled by covered entities. PIPEDA focuses on consent-based use and applies to both health and non-health personal information within Canada’s private sector [6]. In this article, we frame patient information in accordance with the HIPAA definition of PHI, while recognizing that the collaborative steps toward a balanced data governance framework are applicable across different regulatory contexts.

When it comes to imaging data, more sophisticated approaches to de-identification are required because PHI can be burned directly into the images. Data de-identification for Digital Imaging and Communications in Medicine (DICOM) files should capture both pixel-based data and metadata, which are text or numerical values under individual DICOM Tags. Specialized procedures and optical character recognition (OCR) software are necessary to evaluate pixel-based data for proper de-identification of medical images. The identified characters that contain PHI are then masked, usually by covering them with black boxes. Data de-identification tools have achieved de-identification success rates of more than 90% for DICOMs [7]. More recently, large language models (LLMs) are also being explored for their potential in de-identifying medical data [8]. The expectation is that LLMs will further improve the accuracy of DICOM de-identification, particularly by enhancing contextual accuracy, i.e., correctly flagging PHI in text by analyzing its context. A recent study applying LLMs for clinical text de-identification achieved a success rate above 99% in removing personal identifiers from text [8]. However, potential limitations for LLMs include biases in training data, hallucinations, high computational costs, and the need to integrate LLMs with image processing techniques to ensure that visible identifiers in images (e.g., text in images, patient-specific markers) are also properly redacted.

Considering organizations have access to de-identification tools, data sharing decisions should factor in additional considerations. In Box 1, we list principles that healthcare organizations should discuss in their data governance bodies, such as data access committees or ethics review boards, where stakeholders from legal, clinical, technical, and patient advocacy backgrounds can be represented. We recommend that these conditions be carefully evaluated to guide data-sharing decisions across different contexts. The case for facilitating data sharing becomes more compelling when all the listed conditions are met.

**Box 1. Key factors to consider for medical imaging data sharing decisions**

- The risk of re-identifying individuals is low, even when combined with other available datasets.
- Access to the shared data is controlled by defined conditions, including data licensing agreements covering the distribution of data and terms of use.
- There is no special interest or incentive for parties to attempt re-identification.
- The data will be used for a healthcare-related purpose, such as medical research.
- The repurposing of data serves a greater societal good, like preventing diseases or reducing the burden of health problems.

Healthcare systems in general do not support full data transparency with patients nor promote comprehensive data-sharing mechanisms involving patient participation. Previous research indicates that the lack of transparency is due to a combination of factors, including organizational concerns about additional workload and disrupted workflows, and a lack of understanding of the legal framework around data sharing [9,10]. However, our empirical observation is that healthcare organizations are increasingly interested in contributing to medical advancements that depend on data. Involving patients in data governance discussions will help mitigate concerns about data misuse, increase credibility in the decision-making process, and potentially increase participants in data-driven research. Patient education efforts are crucial, along with greater transparency about how their data is used. Healthcare organizations should provide clear, comprehensible information about the intended use of patient data, employing accessible language, visual aids such as infographics, and frequently asked questions (FAQs). To facilitate transparency, a practical approach involves the development of dashboards or platforms that allow the public to track how their data is being used, which research initiatives it supports, and the associated outcomes. Feasibility is supported by existing tools such as the MyData initiative, which enables individuals to manage access to their personal information, and FHIR-based patient portals and apps, which allow secure retrieval and sharing of medical records across systems [11]. These efforts foster trust and demonstrate the value of data sharing. Furthermore, collaborating with trusted third-party entities or data intermediaries, who specialize in managing sensitive data, can ensure adherence to privacy regulations and governance standards. These intermediaries can function as neutral actors, aggregating, de-identifying, and distributing data on behalf of patients or healthcare organizations while maintaining compliance with relevant privacy protections. In formulating the governance recommendations presented here, care was taken to avoid the influence of potential conflicts of interest by grounding the proposals in widely accepted ethical principles. Multidisciplinary perspectives—spanning legal, clinical, technical, and patient advocacy viewpoints—were taken into account to ensure balanced and inclusive guidance. Potential conflicts of interest related to data management are disclosed in the Competing Interests section.

One example of greater patient involvement in data sharing is the UK Biobank project, which collects health data from 500,000 individuals. UK Biobank participants have specific rights that ensure ethical oversight and transparency, including broad informed consent, the ability to withdraw at any time, and guarantees that their data is used only for approved health-related research serving the public interest. While participants do not make direct decisions, their perspectives are represented by the independent Ethics and Governance Council (EGC), which oversees UK Biobank’s practices to ensure alignment with participant values and expectations. The EGC regularly reviews policies, audits data access procedures, and advises on emerging ethical issues, effectively acting as a proxy for participant interests. Transparency is further supported through publicly available governance documents, newsletters, and ongoing communication with participants about data use [12]. To date, more than 16,000 peer-reviewed publications have used UK Biobank data; abstracts and reference details are publicly available on their website [13]. Research has led to discoveries in fields such as dementia, cardiovascular disease, and metabolic health. The biobank’s imaging data include brain, cardiac, abdominal, and musculoskeletal MRI, as well as eye imaging datasets.

In conclusion, imaging data offers great potential for advancing medical research, but its governance requires tailored approaches to ensure patient privacy is not compromised. Data governance frameworks must find a balance between protecting individual privacy and enabling research that can deliver meaningful health benefits. Data sharing decisions should factor in relevant considerations such as the risk of re-identification, the purpose of data use, and the application of data licensing terms. Explaining the benefits, risks, and opportunities of data sharing to patients and society at large is paramount to increasing trust and elevating their stake in data sharing decisions.
